# The Effect of Consumption of Citrus Fruit and Olive Leaf Extract on Lipid Metabolism

**DOI:** 10.3390/nu9101062

**Published:** 2017-09-26

**Authors:** Nicola Merola, Julián Castillo, Obdulio Benavente-García, Gaspar Ros, Gema Nieto

**Affiliations:** 1 Department of Food Technology, Nutrition and Food Science, Veterinary Faculty University of Murcia, Campus de Espinardo, 30100 Espinardo, Murcia, Spain; nicola_merola@um.es (N.M.); gros@um.es (G.R.); 2 Research and Development Department of Nutrafur-Frutarom Group, Camino Viejo de Pliego s/n, 80320 Alcantarilla, Murcia, Spain; j.castillo@Nutrafur.com (J.C.); o.benavente@Nutrafur.com (O.B.-G.)

**Keywords:** olive and citrus extract, cholesterol, flavonoids, phenolics, oleuropein, FOT—Fat Oral Test-, weight control

## Abstract

Citrus fruit and olive leaves are a source of bioactive compounds such as biophenols which have been shown to ameliorate obesity-related conditions through their anti-hyperlipidemic and anti-inflammatory effect, and by regulating lipoproteins and cholesterol body levels. Citrolive™ is a commercial extract which is obtained from the combination of both citrus fruit and olive leaf extracts; hence, it is hypothesised that Citrolive™ may moderate metabolic disorders that are related to obesity and their complications. Initially, an in vitro study of the inhibition of pancreatic lipase activity was made, however, no effect was found. Both preliminary and long-term evaluations of Citrolive™ on lipid metabolism were conducted in an animal model using Wistar rats. In the preliminary in vivo screening, Citrolive™ was tested on postprandial plasma triglyceride level after the administration of an oil emulsion, and a significant reduction in postprandial triacylglycerol (TAG) levels was observed. In the long-term study, Citrolive™ was administered for 60 days on Wistar rats that were fed a high-fat diet. During the study, several associated lipid metabolism indicators were analysed in blood and faeces. At the end of the experiment, the livers were removed and weighed for group comparison. Citrolive™ treatment significantly reduced the liver-to-body-weight ratio, as supported by reduced plasma transaminases compared with control, but insignificantly reduced plasma low density lipoprotein (LDL) and postprandial TAG plasma levels. In addition, faecal analysis showed that the treatment significantly increased total cholesterol excretion. On the other hand, no effect was found on faecal TAG and pancreatic lipase in vitro. In conclusion, treatment ameliorates liver inflammation symptoms that are worsened by the effects of high fat diet.

## 1. Introduction

Obesity is a worldwide metabolic dysfunction that is characterised by an accumulation of excessive amounts of body fat and it is associated with the onset of several pathological conditions such as type 2 diabetes, coronary heart disease, steatosis, and dyslipidemia [1,2]. In today’s increasingly overweight society, the problems that are associated with excess caloric intake are well recognised. In animal models, excessive consumption of dietary fat causes a strong inhibition of lipogenesis by altering both blood hypertriglyceridemia and hepatic lipid levels [3]. These physiological effects are further exacerbated by uncontrolled diabetes mellitus, obesity, and a sedentary lifestyle [4]. In order to treat obesity, several therapeutic strategies have been developed to fight the worldwide epidemic. One of these is focused on the inhibition of the pancreatic lipase (PL) enzyme [5]; another, by decreasing blood cholesterol and triacylglycerol (TAG) levels; and finally, another strategy focuses on blood lipoprotein level management [6].

In this sense, there is a tendency towards searching for new extracts with biologically active components and potential health-promoting properties, including the potential to prevent obesity [6,7]. Phenolics and biophenols are compounds which are formed during plant secondary metabolism and are widely present in the plant kingdom. They are distributed into several classes, i.e., flavonoids (flavanones, flavones, flavonols, isoflavones, flavan-3-ols, anthocyanins, etc.), lignans, stilbenes, terpenoids, iridoids, caffeoyl compounds, and other phenolic derivatives, which are distributed in plants and food of plant origin [8].

The extract presented in this study is called ‘Citrolive™’ and it is obtained from the combination of olive iridoids (oleuropein family) and citrus flavonoids. These chemical classes are being increasingly studied for their influence on lipid metabolism. For example, the European Foods Safety Authority (EFSA) has issued a scientific opinion stating that the consumption of biophenols from olives can be advertised with claims regarding the protection of low density lipoprotein (LDL) particles from oxidative damage, as well as the maintenance of a normal concentration of blood high density lipoprotein (HDL) and cholesterol [9]. In addition, it has been reported that olive extracts from the leaf and fruit have been shown to possess utility as an obesity management tool [10]. Regarding citrus fruits, it has been suggested that flavonoids, the main compounds that are present in citrus, are associated with a reduced risk of cardiovascular disease and possess anti-inflammatory proprieties [11]. In addition, it has been observed that flavonoids possess health-promoting benefits, improving cholesterol levels in rats that were fed high-fat diets [12]. Although studies on flavonoids and biophenols have demonstrated their bioactivity in the management of lipid metabolism, no studies have been carried out to test their chemical combination on TAG, lipoprotein, or lipid metabolism management. This research may lend further support for the employment of the extract as an obesity prevention tool.

Taking into account the health benefits of the compounds that are present in the extract, and the lack of information about the effects of Citrolive™ on lipid metabolism, in the present study, two independent studies were carried out: a short-term experiment and a long-term experiment. The former included a pancreatic lipase inhibition activity test in vitro, and a postprandial TAG level test in vivo. The latter evaluated the chronic administration effects of Citrolive™ over 60 days. Wistar rats were employed in both experiments as a biomodel. In the long-term study, they were fed with a high-fat diet in order to induce obesity. Finally, several clinical parameters related to obesity status were evaluated.

In order to elucidate these findings, the aim of the present study was to ascertain the effect of a combination of citrus fruit and olive leaf extract consumption on lipid metabolism in rats with a high-fat diet with induced obesity in order to identify the effect of Citrolive™ intake on the management of lipid metabolism.

## 2. Material and Methods

### 2.1. Reagents and Chemicals

Virgin olive oil was obtained from a local market and was employed without further purification (Hacendado, Spain); egg yolk lecithin was obtained from Sigma-Aldrich (St. Louis, MO, USA), capillary tubes from Sarsted (CB 300-microvette, Germany), and isofluorane (Baxter, Germany). Two commercial presentations of orlistats were used in the experiment: a pharmaceutical orlistat presentation from Alli^®^ (Spain) was used in oral tests in rats, while the compound that was obtained from Sigma (O4139, St. Louis, MO, USA) was used in the in vitro pancreatic lipase activity test. High-performance liquid chromatography (HPLC) standards (i.e., naringin, neohesperidin, oleuropein, and hydroxytyrosol) were obtained from Extrasynthèse (Genay, France); all of the reagents that were employed in the analyses were of HPLC grade and were supplied by Sigma (Madrid, Spain).

### 2.2. Citrus Fruit and Olive Leaf Extract (Citrolive^TM^)

Citrolive™, the natural extract that is used in this study, was obtained from Nutrafur S.A-Frutarom Group (Murcia, Spain) and was used without further processing. All of the other chemicals that were used in the study were of the highest commercial grade available.

### 2.3. High-Performance Liquid Chromatography (HPLC) Conditions

The HPLC equipment that was used was a Hewlett-Packard Series HP 1100 that was equipped with a diode array detector. The stationary phase was a C_18_LiChrospher 100 analytical column (250 × 4 mm i.d.) with a particle size of 5 nm (Merck, Darmstadt, Germany) thermostated at 25 °C.

For the elucidation and quantification of bioactive compounds in the Citrolive™, the extract was dissolved in dimethylsulfoxide (DMSO) at a ratio of 5 mg/mL and this solution was filtered through a 0.45-nm nylon membrane. The flow rate was 1 mL/min and the absorbance changes were monitored simultaneously at 280 and 340 nm. The mobile phases for chromatographic analysis were as follows: (A) acetic acid/water (2.5:97.5) and (B) acetonitrile. A linear gradient was run from 95% (A) and 5% (B) to 75% (A) and 25% (B) during the first 20 min; which changed to 50% each of (A) and (B) after another 20 min (40 min in total); which, after 10 more minutes, changed to 20% (A) and 80% (B) (50 min in total), and finally equilibrated over the last 10 min (60 min in total) to the initial composition.

### 2.4. Short-Term Study In Vitro (PL Inhibition) and In Vivo (Fat Oral Test)

#### 2.4.1. Short-Term Study: Citrolive™ Inhibition of Pancreatic Lipase Activity (PL) In Vitro Assay

The amount of inhibition of lipase activity was determined using a Bioteck plate reader by measuring the amount of 4-metyhl-umbelliferone-oleate (4-MU-oleate) product that was released by porcine PL. The method that was employed was adapted from Nakai et al. [13] with small modifications. Briefly, 25 µL of Citrolive™ or orlistat were dissolved in DMSO, and 25 µL of 0.1 mM 4-MU-oleate was dissolved in 100 µL 13 mM tris-HCl, 150 mM NaCl, and 1.3 mM CaCl2 at a pH of 7.8. The reaction was started by adding 50 µL of fresh porcine pancreatic lipase (50 U/mL). The 96-well microplate was read at 25 °C for 10 min at 20-s intervals at an excitation of 355 nm and an emission of 460 nm. The 50% inhibitory concentration (IC50) of each test sample was obtained from the least-squares regression line plots of the logarithm of the sample concentrations (*X* = log[*X*]) versus the normalized pancreatic lipase activity (%). The experiment was carried out in triplicate.

#### 2.4.2. Short-Term Study: Citrolive™ Postprandial TAG Levels (Fat Oral Test) In Vivo Assay

##### Animals

**Figure 1:**
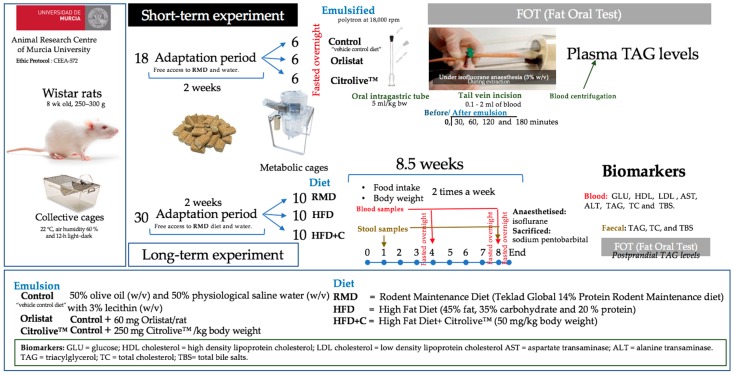
Study design of short- and long-term experiments to evaluate the effect of the consumption of citrus fruit and olive leaf extract on lipid metabolism.

Eighteen 8-week-old male Wistar rats, weighing 250–300 g, were provided by the Animal Research Centre of Murcia University. The rats were maintained under controlled temperature (22 °C), air humidity (60 ± 5%), and light–dark cycle (12-h) conditions for 2 weeks (adaptation period) with free access to the standard laboratory diet (standard laboratory diet, Rodent Maintenance Diet, RMD, Teklad Global 14% Protein Rodent Maintenance diet, Harland Laboratories) and water ad libitum before starting the experiment. Animals were randomly divided into three groups (Control, Orlistat, and Citrolive™ diet) and placed individually in metabolic cages under the same environmental conditions described above (Figure 1).

##### Experimental Design

The postprandial triglyceride level’s procedure was carried out according Merola et al. [14]. Animals were divided into three groups (six in each group) and fasted overnight (Figure 1). Afterwards, each group was orally administered an emulsion with different dietary compositions. The group ‘Control’ was administered a “vehicle control diet” that consisted of 50% olive oil (*w*/*v*) and 50% physiological saline water (*w*/*v*) with 3% lecithin (*w*/*v*). The second group, ‘Orlistat’ [15], was treated with a “vehicle control diet” plus orlistat, which was solubilised prior the emulsification step at 60 mg/rat. The third group, ‘Citrolive’, was administered a “vehicle control diet” plus Citrolive™ extract, which was solubilised prior to the emulsification step at a concentration of 250 mg/kg body weight. Every diet was emulsified with a polytron at 18,000 rpm and then orally administered to the rats via an intragastric tube at a concentration of 5 mL/kg body weight, always within 30 min from preparation. Blood samples were collected via a tail vein incision before the oral administration, and at 30, 60, 120, and 180 min thereafter. The procedure was carried out under isofluorane anaesthesia (3% *w/v*), which was used only during the blood collection procedure. Finally, the blood was centrifuged so that plasma and TAG levels could be measured with a commercial colorimetric assay (Olympus triglyceride OSR6133) in an Olympus AU600 autoanalyser (Olympus, Tokyo, Japan). The technique is based on a series of combined enzymatic reactions with the formation of a product with a maximum absorbance at 500 nm. Finally, the TAG in the samples was measured proportionally with the rise of the absorbance until 520/600 nm. Plasma TAG was expressed incrementally from the baseline. Incremental areas under the response curves (AUC) during the whole time under study were calculated using the trapezoidal rule, with fasting levels as the baseline. The animal study was carried out under appropriate guidelines and was approved by the Bioethics Committee of Murcia University (authorization number: CEEA-572).

### 2.5. Long-Term Study: Citrolive™ Chronic Administration during 60 Days in Wistar Rats

#### 2.5.1. Animals

Thirty 8-week-old male Wistar rats, weighing 250–300 g, were provided by the Animal Research Centre of Murcia University. The rats were maintained under controlled temperature (22 °C), air humidity (60% ± 5%), and light–dark cycle conditions (12-h each) for 2 weeks before starting the experiment (Figure 1). Water and RMD were provided ad libitum. In order to mimic overweight-obesity, animals were fed with hypercaloric diet (high-fat diet, HFD, RMD with 45% fat, 35% carbohydrate, and 20% protein, D12451 research diet; New Brunswick, USA). Animals were divided into three groups (10 rats each): the first group was fed with RMD, the second with a HFD, and the third with the HFD plus Citrolive™ extract (60 mg/kg body weight, the same dose as that of orlistat) (HFD + C). The animal study was carried out under appropriate guidelines and was approved by the Bioethics Committee of Murcia University as mentioned above.

#### 2.5.2. Experimental Design

The experimental period was 8.5 weeks, and during this time, the animals were given free access to feed and drink. Food intake and body weight were measured twice a week (Figure 1). Blood samples were collected before the experiment and at 30 and 60 days after overnight fasting. Stool samples were collected at 7 and 60 days. At the end of the experiment, all rats were deprived of food overnight, anaesthetised with isoflurane, and sacrificed using an intraperitoneal injection of sodium pentobarbital. Livers were collected from the 30 animals as biological samples and weighed with an analytical balance. Livers were immediately cut into small pieces and then frozen with liquid nitrogen. Liver samples were stored at −80 °C until the analytical procedures were carried out.

### 2.6. Anthropometrical Analysis

Body length (nose-to-anus length) was determined in all of the groups at the beginning and at the end of the experiments. The measurements were made on anaesthetized rats (3% *w*/*v* isofluorane). The body weight and body length were used in order to determine the body mass index according to the following formula: Body mass index (BMI) = Body weight (g)/Length^2^ (cm^2^).

### 2.7. Blood Sampling and Analytical Biomarkers

Blood samples were transferred into heparin-containing tubes. Plasma was obtained by centrifugation (3000 rpm, 10 min, 4 °C). Glucose (GLU), total triglycerides, total cholesterol, HDL cholesterol and LDL cholesterol, and the activity of aspartate transaminase (AST) and alanine transaminase (ALT) enzymes were analysed in plasma samples using an automatic analyser (AU 600 Olympus Life, Hamburg, Germany). All analyses were performed in triplicate. The liver-to-body-weight ratio was calculated according to the following formula: Liver weight (g)/Body weight (g).

### 2.8. Faecal Extraction and Faeces Measurements and Analyses

Faecal fat extraction was adapted from Argmann et al. [16]. Briefly, the rats were placed in clean cages containing a metal floor grid instead of bedding. Faeces were collected over a 24-h period in parallel with a food intake measurement in order to determine the fat balance (lipid intake and output). The collected faeces were dried for one hour in a thermostatic oven at 70 °C and weighed using an analytical balance. For the extraction, a 2:1 chloroform/methanol solution was added to 500 mg of dry stools and homogenised for ~30 s at a high speed in a polytron-type homogeniser. Then, methanol was added and the tubes were centrifuged for 15 min at 750× *g*, room temperature. After removing the supernatant chloroform, 0.73% NaCl solution was added and the samples were centrifuged again for another 3 min at 750× *g*, room temperature. The top phase was discarded and the phase interface was washed three times adding a chloroform/methanol/NaCl solution. Finally, the samples were evaporated to dryness under a steady stream of nitrogen and were then re-suspended in deionised water. Faecal TAG, TC, and TBS values were taken after 5 and 60 days from the beginning of the experiment and were analysed with the same reagents that were used for plasma analysis.

### 2.9. Postprandial TAG Levels (Oral Fat Test) In Vivo Assay

Postprandial TAG levels were assessed according to the same protocol that was used in the preliminary assay with small modifications. In order to evaluate the postprandial TAG systemic level curve at 30 and 60 days, the first group, HDF (*n* = 6), was administered the vehicle control diet as described in the preliminary study assay; the other group, HDF + C (*n* = 6), was administered the vehicle control diet plus Citrolive™ extract and the animals that did not receive the vehicle control or the treatment were administered with physiological saline. Afterwards, the vehicle control, vehicle control plus extract, and physiological saline were orally administered via an intragastric tube at 5 mL/kg body weight. Blood samples were obtained by tail incision at 120 and 180 min after the administration of the emulsion.

### 2.10. Statistical Analysis

The statistical analyses in the classical biomarkers were carried out using GraphPad statistical software, and the results are expressed as the mean ± SD (standard deviation). A two-way analysis of variance (ANOVA) followed by Dunnett’s post-hoc tests were applied to determine the differences for all variables among the groups (RMD mean as control). A paired Student’s *t* test was also carried out to ascertain the significant differences of means in clinical analyses of blood and faeces between groups (HFD and HFD + C) at each point in time. The threshold *p* value chosen for statistical significance was *p* < 0.05.

## 3. Results

### 3.1. Polyphenolic Distribution: HPLC Analysis

The phenolic compounds in Citrolive™ were screened and their contents were analysed by HPLC. The abundance (absolute content, on an as is basis, *w*/*w*) of the main compounds in Citrolive™ has been fully described in Table 1. Five groups of compounds are principally present in Citrolive™: from olive leaves origin: secoiridoids (oleuropein), phenolics (hydroxytyrosol, tyrosol, vanillic acid, and caffeic acid), and polyphenols (verbascoside), and from citrus origin: flavanones (naringin, neohesperidin, neoeriocitrin, and hesperidin) and flavones (luteolin-7-glucoside, apigenin-7-glucoside, diosmetin-7-glucoside, luteolin, and diosmetin).

### 3.2. Short-Term Study: Citrolive™ Inhibition of Pancreatic Lipase Activity (PL) In Vitro Assay

The IC_50_ of Citrolive™ on PL inhibition is expressed in µg/mL and the related confidence interval (CI) is the following: while orlistat had an IC_50_ of 0.04 µg/mL (CI of 0.04 to 0.05), Citrolive™ had an IC_50_ of 70.92 (CI of 51.41 to 97.82). The solvent (DMSO) alone had no effect on PL activity.

### 3.3. Short-Term Study: Citrolive™ Postprandial TAG Levels (FOT—Fat Oral Test) In Vivo Assay

**Figure 2:**
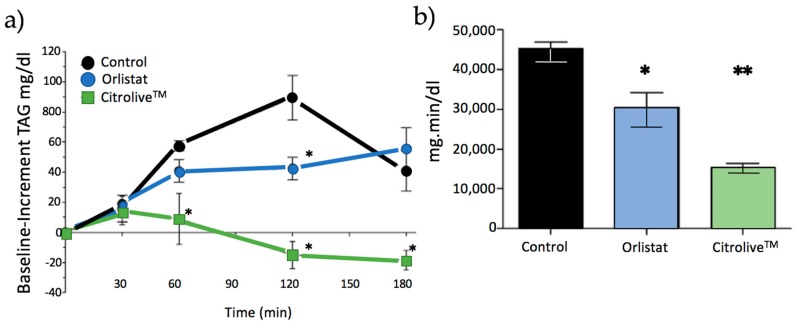
(**a**) Postprandial plasma TAG levels of the different oil-based emulsion vehicles during 180 min after their administration. The values were expressed as increment from basal TAG values (TAG mg/dL increment mean ± SD, *n* = 6). Stars indicate that the values were significantly different (*p* < 0.05 with *); (**b**) Area under the curve (AUC) of the test compounds. Stars indicate that the values were significantly different (*p* < 0.05 with *; *p* < 0.01 with **).

The postprandial TAG response is shown in Figure 2a. The plasma TAG level in the control group exhibited a constant increase until reaching a postprandial peak at 120 min; then it started decreasing at 180 min. Orlistat started to increase the TAG level after 60 min, and the latter remained constant until 120 min had passed; it reached significant values at 120 min, reducing the TAG level by almost 50% (*p* < 0.05). Citrolive™ extract significantly reduced TAG increment levels (*p* < 0.05) at all of the time points that were taken into account (except at 30 min). The AUC (area under curve) for TAG response is reported in Figure 2b. Citrolive™ had a major effect towards reducing plasma TAG levels (*p* < 0.01). This effect was even stronger than the one that was produced by orlistat (*p* < 0.05).

### 3.4. Long-Term Study: Citrolive™ Chronic Administration during 60 Days in Wistar Rats

During the experiment, no abnormal clinical signs in the rats were observed. Feeding rats with a HFD resulted in a significant body weight increment compared with the RMD by the end of the experiment (Table 2). Although no difference was found between the HFD and HFD + C groups, the treatment resulted in a 4% body weight reduction. BMI was not different between the HFD and treatment groups, but both showed differences when compared with the RMD group (*p* < 0.05). The mean food intake per week per rat was significantly different (*p* < 0.01) between the RMD and HFD diet groups (255 ± 33 kJ in the RMD group and 356 ± 52 kJ in the HFD group). No difference was found between the HFD and HFD + C diet groups (356 ± 52 kJ in the HFD group and 376 ± 47 kJ in the HFD + C group).

### 3.5. Clinical Measurements

**Figure 3:**
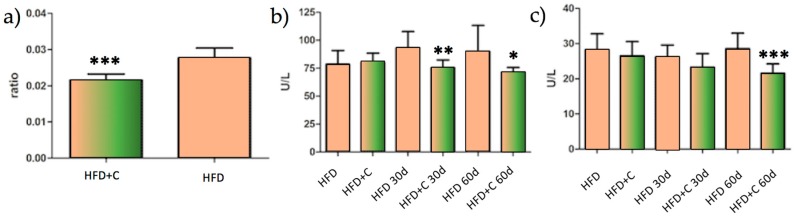
(**a**) Liver-to-body-weight ratio comparison between HFD and HFD + C groups; (**b**) HFD and HFD + C groups comparison of AST and (**c**) ALT transaminase at the beginning, and 30 and 60 days after the experiment. Stars indicate that the values were significantly different (*p* < 0.05 with *, *p* < 0.01 with **, and *p* < 0.001 with ***, mean ± SD *n* = 10).

A liver-weight-to-body-weight comparison between HFD and HFD + C groups is shown in Figure 3a. The HFD + C group’s liver-to-body-weight ratio was significantly lower compared with the HFD group’s (*p* < 0.001). Biochemical analysis showed that AST and ALT transaminase had lower values in the HFD + C group compared with the HFD group at 30 days of treatment (AST *p* < 0.01). At 60 days, both ALT and AST were significantly lower (AST *p* < 0.05 and ALT *p* < 0.001) (Figure 3b,c). The clinical analysis that was performed on the blood did not suggest a significant difference between the HFD and HFD + C groups for GLU, TC, and HDL (Table 2). However, plasma TAG was reduced by 8% in the HFD + C group at 30 and 60 days, while LDL was reduced by ~20% (*p* < 0.052) at 60 days.

### 3.6. Dry Weight, Total Fat, TAG, Total Bile Salts (TBS), and Cholesterol Analysis in Faeces

**Figure 4:**
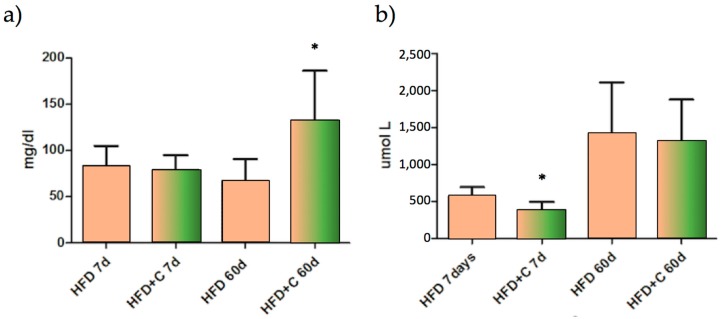
(**a**) Faecal TC showed that values were significantly higher in the HDF + C group compared with the HFD group by the end of the treatment; (**b**) Faecal TBS values comparison between HFD and HFD + C groups. Stars indicate that the values were significantly different (*p* < 0.05 with *, *p* < 0.01 with **, and *p* < 0.001 with ***, *n* = 10).

No diarrhoea was observed throughout the experiment. Faecal fat excretion and dry faeces weight were significantly higher in the HDF + C group compared with the HFD group (*p* < 0.05) (Table 3). Faecal fat analysis showed that TC values were significantly higher in the HDF+C group compared with the HFD group by the end of the treatment (Figure 4a). TBS values were also altered but only at 7 days after treatment (Figure 4b). On the other hand, faecal TAG values were not affected by the treatment (Table 3).

### 3.7. Long-Term Study: Postprandial TAG Levels (Oral Fat Test) In Vivo Assay

**Figure 5:**
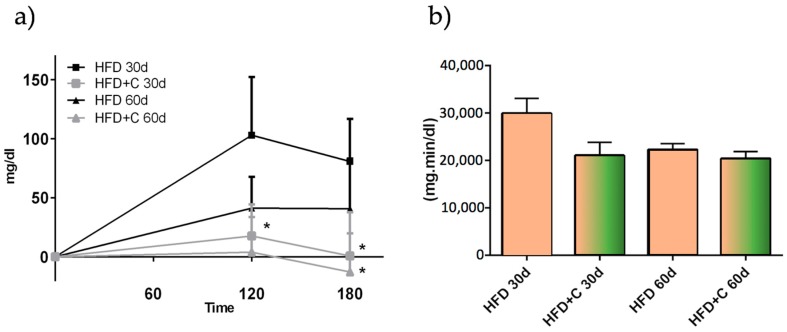
(**a**) Plasma TAG kinetic curve comparison between HFD (black square) and HFD + C (grey triangle) during the experimental study trial. The values were expressed as increment from basal TAG values (TAG mg/dL increment mean ± SD, *n* = 6). Stars indicate that the values were significantly different (*p* < 0.05 with *); (**b**) Area under the curve (AUC) of the experimental groups at different point of times.

As opposed to physiological saline, all of the treatments that were administered orally in oil-based emulsion form altered the plasma TAG kinetic curve during the experimental study trial. As shown in Figure 5a, at 30 days after the beginning of the experiment the extract significantly blunted plasma TAG increase at 120 and 180 min (*p* < 0.05). However, at 60 days, the significance was attained only at 120 min (*p* < 0.05). Although not statistically significant, the AUC HFD + C value was lower compared with the AUC HFD value at 30 (*p* < 0.07) and 60 days (Figure 5b). On the other hand, the HFD group showed a decreased AUC from 30 to 60 days during the experiment. No difference in the TAG plasma kinetic curve and AUC between the groups was found in the animals that were receiving physiological saline solution. 

## 4. Discussion

In this paper, we carried out two studies: a short-term (1 day) and a long-term (60 days) evaluation of Citrolive™ on lipid metabolism. The activity of Citrolive™ was tested short-term towards pancreatic lipase activity in vitro and on TAG plasma levels in vivo. In both tests, orlistat was employed as a positive control, considering that several studies positively associate orlistat treatment with reduced total and low density lipoprotein (LDL) cholesterol levels and postprandial TAG [17]. Therefore, we tested the in vivo postprandial TAG plasma levels, which is a common test for evaluating gastrointestinal lipid malabsorption [18] and is increasingly being employed to corroborate the potential activity of natural extracts as pancreatic lipase (PL) enzyme inhibitors [19].

FOT (short-term study) showed that Citrolive™ reduced postprandial TAG to lower levels than orlistat did (Figure 2). To ascertain the mechanism of action, we tested whether this result was due to an orlistat-like effect of the extract on PL, or to the reduction of TAG systemic production. The key to evaluating the orlistat-like process is to study the activity of the PL, which splits triglycerides into two products (more hydrophilic than their precursors): two molecules of fatty acid and one of 2-monoglyceride. These lipolytic products are still sparingly soluble in the aqueous environment of the intestine, and require bile salts and phospholipids for their incorporation into micelles, which are polymolecular aggregates which act as shuttles, delivering fatty acids and monoglycerides to the intestinal microvilli, where they dissociate from micelles and diffuse inside the enterocyte. Subsequently, intestinal cells re-synthesise triglyceride molecules and incorporate them into chylomicrons which are secreted to the intestinal lymph. However, the IC50 value of Citrolive™ for PL inhibition was negligible compared with the PL inhibitor, thus invalidating the hypothesis that the postprandial TAG plasma reduction effect was due to orlistat-like PL inhibition.

This result, given the need to understand it, led us to carry out a long-term trial in order to evaluate the metabolic consequences of this physiological effect during the chronic administration of the extract in Wistar rats. Interestingly, the main result that was derived from this study is a reduced hepatic injury level from decreasing liver inflammatory parameters in rats that were fed a high fat diet plus Citrolive™ for 60 days. As shown in Figure 3a, the liver-to-body-weight ratio of the Citrolive™ group was 21% lower compared with the high-fat diet control group. In addition, Citrolive™ decreased the liver intracellular enzymes ALT and AST (Figure 3b,c). The former had already displayed significantly lower values at 30 days after the beginning of the experiment.

On the other hand, in the Citrolive™ group, the LDL concentration was reduced in plasma by almost 20% with respect to the control group. This led us to consider that Citrolive™ may reduce intestinal cholesterol uptake, influencing LDL synthesis and clearance, rather than inhibiting synthetic hepatic enzymes. According to cholesterol homeostasis, cholesterol metabolism regulates LDL receptor activities and contributes accordingly to the regulation of serum cholesterol levels [20]. Therefore, the extract that was studied may have influenced intestinal cholesterol uptake, reducing the rate of LDL formation in plasma. A decreased chylomicron cholesterol uptake may have caused hepatocytes to respond by upregulating the LDL receptors on their plasma membranes. This, in turn, decreases LDL concentrations by facilitating the clearance of LDL particles from the plasma [21]. Consequently, these results suggest that one of the mechanisms whereby Citrolive™ extracts beneficially reduce hepatic inflammation in rats that are fed it is by reducing intestinal cholesterol absorption.

Lipid faecal analysis showed that, compared with the control, faecal total fat was increased in the Citrolive™ treatment and that this effect was significantly (*p* < 0.05) associated with the high content of TC (Figure 4a). However, this parameter was not affected by plasma TC, so faecal hypercholesterolemia is not dependent upon reduced hepatic synthesis or increased total faecal biliary salts (Figure 4b).

As mentioned previously, postprandial TAG plasma uptake alteration is a process that may have contributed towards reducing hepatic inflammation, as evidenced by the reduced blood postprandial TAG level after the administration of an oral emulsion. In addition, a TAG decrement might have increased faecal TAG; however, in this study, TAG excretion in faeces was not significantly altered by the treatment. Another process that might have affected TAG absorption is reduced TAG production in the liver. With good pancreatic function enabling the efficient hydrolysis of dietary TAG and normal biliary function translating into a correct micellar solubilisation of lipolytic products, the healthy intestine has a great capacity to absorb a dietary fat load in such a way that up to 95% of the fatty acids and monoglycerides are absorbed and the faecal fat content is usually below 5% [22]. On the other hand, a diminished activity of the pancreatic lipase enzyme would increase the unmetabolised faecal TAG concentration, and will decrease plasmatic TAG values.

This effect was not due to diminished plasma TAG absorption since AUC values were not affected by the Citrolive™ treatment, and the plasma TAG in Citrolive™-treated rats in the long-term experiment was reduced by only 8% compared with the high-fat diet group (Figure 5). Consequently, regarding the Citrolive™ in vivo study, and considering that the extract did not significantly affect body weight and food intake throughout the experiment, the mechanism by which Citrolive™ reduced plasma TAG levels remains elusive.

Citrolive™ extract’s composition is shown in Table 1. According to HPLC analysis, oleuropein accounts for more than 15% of the total extract, which was its most prevalent compound. The study of Hur et al. [23] determined in mice whether oleuropein shows a protective effect against hepatic steatosis that was induced by a high-fat diet in order to elucidate its underlying molecular mechanisms, along with several key transcription factors. It was also studied whether their target genes were involved in adipogenesis, and whether they were downregulated by oleuropein. The conclusion of these authors is that oleuropein decreased the number and size of lipid droplets in free fatty acid treated cells, and reduced intracellular triglyceride accumulation. It is interesting that, in accordance with our results, the protective effects of oleuropein were against Free fatty acids (FFA)-induced hepatocellular steatosis, and the effects that are shown in both studies may be explained by the mechanism of oleuropein [23,24].

Another mechanism by which oleuropein may have reduced inflammation is through interference with some digestive enzymes other than pancreatic lipase. In this sense, in a study by Polzonetti et al. [25], oleuropein was shown to modulate some digestive enzymes like pepsin and trypsin. Future findings are in progress to evaluate and quantify the possible synergistic contribution of citrus flavonoids to each one of these mechanisms.

As above mentioned, hepatic injury tends to be associated with the development of inflammation, progressive metabolic dysregulation, and steatosis (fatty infiltration of the liver leading to cirrhosis and hepatocellular carcinoma). This reflects a difference between the rate at which fatty acids reach hepatocytes and the rate at which they are metabolised, stored, or assembled [26].

Out of all these findings we hypothesise that Citrolive™ treatment reduced inflammation and the accumulation of TAG in the steatotic liver through different physiological processes, namely increasing faecal total cholesterol values and altering postprandial TAG plasma uptake.

However, as whole, the results that were obtained from this study did not offer a clear answer regarding the alteration of plasma postprandial TAG uptake. Since intestinal TAG digestion is a complicated process, including phases such as emulsification, the hydrolysis of fatty acid ester bonds by specific esterases, the aqueous dispersion of lipolytic products in bile acid micelles, and absorption, mainly in the proximal jejunum but also in more distal parts of the small intestine, in order to explain our study results we have to look more carefully into these digestion processes.

## 5. Conclusions

In conclusion, Citrolive™ treatment ameliorates liver inflammation symptoms worsened by the negative effects of high fat diet. This was demonstrated by lower liver to body weight, decreased ALT and AST transaminases, increased faecal TC, and altered postprandial TAG plasma uptake. From the study of the extract composition, oleuropein is reputed to be one of the main candidates to exhibit those effects, by influencing systemic anti-inflammatory capacity and digestive enzyme activity. Citrus flavonoids may exert a form of background influence, and this should be evaluated in future studies 

## Figures and Tables

**Table 1:** Absolute content (% *w*/*w*, according to the corresponding standards) of the main bioactive compounds classified by main chemical family and plant origin. Other bioactives are trace, less than 0.1%.

Olive Leave						Citrus			
Secoiridoids		Phenolics		Polyphenols		Flavonoid		Flavonoid	
						Flavanones		Flavones	
Oleuropein	15.74	Hydroxytyrosol	0.84	Verbascoside	0.62	Naringin	3.89	Apigenin 7-O-glucoside	0.82
		Vanillic acid	0.35			Neohesperidin	1.93	Luteolin 7-O-glucoside	0.52
		Tyrosol	0.24			Hesperidin	0.61	Diosmetin 7-O-glucoside	0.31
		Caffeic acid	0.21			Neoeriocitrin	0.41	Luteolin	0.15

**Table 2:** Morphometric parameters and blood biomarkers of the experimental groups (mean ± SD, *n* = 10).

Diet */Time (Day of Sampling)						
	RMD		HFD		HFD + C	
Morphometric parameters	0	60	0	60	0	60
Weight (g)	352.70 ± 29.32 ^b^	453.45 ± 28.00 ^a^	350.25 ± 30.66 ^b^	493.00 ± 42.90 ^a^	351.33 ± 26.82 ^b^	487.33 ± 30.96 ^a^
BMI	0.61 ± 0.03 ^a^	0.62 ± 0.02 ^a^	0.62 ± 0.04 ^a^	0.67 ± 0.04 ^a^	0.76 ± 0.06 ^a^	0.75 ± 0.02 ^a^
Blood biomarkers						
GLU (mg/dL)	85.01 ± 8.12 ^a^	85.75 ± 6.71 ^a^	84.53 ± 9.26 ^b^	151.73 ± 8.85 ^a^	86.81 ± 13.65 ^b^	148.96 ± 16.45 ^a^
TAG (mg/dL)	86.00 ± 12.00 ^a^	81.20 ± 13.30 ^a^	91.10 ± 15.30 ^a^	102.8 ± 25.09 ^a^	87.30 ± 19.90 ^a^	94.08 ± 18.93 ^a^
TC (mg/dL)	61.10 ± 13.52 ^a^	59.15 ± 10.21 ^a^	58.04 ± 13.53 ^a^	56.18 ± 10.21 ^a^	57.26 ± 14.12 ^a^	54.44 ± 3.57 ^a^
TBS (µmol/L)	5.98 ± 1.74 ^a^	6.59 ± 1.35 ^a^	5.85 ± 1.74 ^a^	1.96 ± 0.41 ^b^	5.80 ± 1.97 ^a^	2.53 ± 0.81 ^b^
HDL (mg/dL)	44.19 ± 5.83 ^a^	39.70 ± 6.53 ^a^	43.37 ± 6.30 ^a^	37.24 ± 4.66 ^a^	39.70 ± 6.53 ^a^	35.33 ± 9.27 ^a^
LDL (mg/dL)	13.04 ± 3.34 ^a^	17.00 ± 4.03 ^a^	13.48 ± 2.94 ^b^	19.51 ± 1.97 ^a^	14.21 ± 3.31 ^a^	17.66 ± 2.01 ^a^

* Diet: RMD = Rodent Maintenance Diet; HFD = High Fat Diet; HFD + C = High Fat Diet + Citrolive™; Morphometric parameters: BMI = body mass index Blood biomarkers: GLU = glucose; HDL = high density lipoprotein cholesterol; LDL = low density lipoprotein cholesterol; AST = aspartate transaminase; ALT = alanine transaminase; TAG = triacylglycerol; TC = total cholesterol; TBS = total bile salts; ^a,b^ Values not shearing the same superscript within the same diet and parameter or biomarker are significantly different for *p* < 0.05.

**Table 3:** Faecal analysis of the experimental groups (mean ± SD, *n* = 10).

Diet */Time (Day of Sampling)						
	RMD		HFD		HFD + C	
Faecal analysis	5	60	5	60	5	60
• Dry weight (g)	1.91 ± 0.18 ^a^	1.52 ± 0.41 ^a^	1.76 ± 0.63 ^a^	1.36 ± 0.34 ^a^	2.13 ± 0.39 ^a^	1.65 ± 0.25 ^a^
• Total fat (mg/g)	6.11 ± 13.52 ^a^	5.91 ± 10.21 ^a^	8.33 ± 3.14 ^a^	6.00 ± 2.10 ^a^	7.33 ± 2.07 ^a^	8.55 ± 1.48 ^a^
Biomarkers in faeces						
• TAG (mg/dL)	289.00 ± 29.70 ^a^	391.00 ± 117.10 ^a^	572.00 ± 123.80 ^b^	1411.00 ± 697.80 ^a^	388.30 ± 104.90 ^b^	1313.00 ± 564.50 ^a^
• TBS (µmol/L)	7.97 ± 1.72 ^b^	11.83 ± 2.48 ^a^	19.21 ± 4.73 ^b^	25.17 ± 9.26 ^a^	19.83 ± 4.40 ^a^	18.98 ± 7.80 ^b^

* Diet: RMD = Rodent Maintenance Diet; HFD = High Fat Diet; HFD + C = High Fat Diet + Citrolive™; Biomarkers in faeces: TAG = triacylglycerol; TBS = total bile salts; ^a,b^ Values not shearing the same superscript within the same diet and parameter or biomarker are significantly different for *p* < 0.05.
